# Residue Gln370 of Newcastle disease virus nucleoprotein mediate viral inclusion body-like structure formation via interaction with residue Ser254 of viral phosphoprotein

**DOI:** 10.3389/fcimb.2026.1978310

**Published:** 2026-10-23

**Authors:** Wenbin Wang, Sa Xiao, Yuxia Zhang, Xiaoyuan Yuan, Wenwen Dong, Zhuoming Qin, Feng Hu, Kexiang Yu, Dzmitry Barysavets, Nasonau Ihar, Shchemialiova Natallia, Hongliang Wang, Guiming Li

**Affiliations:** 1 Poultry Institute, Shandong Academy of Agricultural Science, Jinan, China; 2 Jinan Key Laboratory for Agricultural Experimental Animal and Comparative Medicine, Jinan, China; 3 Shandong Provincial Key Laboratory of Livestock and Poultry Breeding (PKL2024B15), Shandong Academy of Agricultural Sciences, Jinan, China; 4 College of Veterinary Medicine, Northwest A&F University, Yangling, China; 5 Institute of Experimental Veterinary Medicine named of S.N. Vyshelessky, Minsk, Belarus

**Keywords:** inclusion body, interaction, Newcastle disease virus, nucleoprotein, phosphoprotein

## Abstract

**Background:**

Newcastle disease virus (NDV) infects more than 250 avian species and mammals. Cytoplasmic inclusion bodies (IBs) are a hallmark of paramyxovirus infection; however, the molecular mechanisms underlying NDV IB formation remain poorly understood.

**Methods:**

In this study, we investigated the interactive sites of viral protein driving IB-like structure formation via transmission electron microscopy, immunofluorescence assays (IFA), viral titration, co-immunoprecipitation (co-IP), structural modeling analysis.

**Results:**

Both virulent and attenuated NDV strains induced IBs in chicken-, mouse-, and human-derived cells. And the virus replicated well in CEF and HeLa cells rather than MEF. Time-course analysis revealed that IBs emerged as small puncta early in infection, increased in size over time, and ultimately accumulated in the perinuclear region. Co-immunoprecipitation (co-IP) and IFA revealed that amino acid residues 367–375 of the nucleoprotein (NP) bound to the PMD of the phosphoprotein (P), and this interaction was required for IB-like structure assembly. Furthermore, structural modeling and experiments of the NP–P complex identified residues Gln370 of NP and residue Ser254 of P as the principal contact and interacting sites for IB-like structure assembly.

**Discussion:**

These findings reveal a previously unrecognized NP–P binding interface that mediates NDV IB formation, providing new insights into the molecular basis of viral condensate biogenesis in paramyxoviruses and a potential target for antiviral intervention.

## 1 Introduction

Newcastle disease virus (NDV), also known as avian paramyxovirus type 1 (APMV-1), is a prototypical member of the genus *Orthoavulavirus* within the family *Paramyxoviridae* and order *Mononegavirales*, which can infect more than 250 species of animals, including poultry and mammals (Kapczynski et al., 2013; Zhou et al., 2025). NDV is an enveloped, pleomorphic virus harboring a single-stranded, nonsegmented, negative-sense RNA genome approximately 15 kb in length (Kapczynski et al., 2013). The NDV genome contains six transcriptional units in the conserved order 3’-NP-P/V/W-M-F-HN-L-5’, encoding six structural proteins: nucleoprotein (NP), phosphoprotein (P), matrix protein (M), fusion glycoprotein (F), hemagglutinin-neuraminidase (HN), and the large RNA-dependent RNA polymerase (L) (Kapczynski et al., 2013; Zhou et al., 2025). Additionally, through an RNA editing process at a specific site within the P gene, NDV produces two nonstructural accessory proteins, V and W, which play critical roles in counteracting host innate immune responses, particularly by antagonizing type I interferon signaling (Kapczynski et al., 2013; Zhou et al., 2025).

Among these viral proteins, NP and P are central to the replication and transcription machinery of NDV. NP serves as the primary scaffold for encapsidation of the viral genomic and antigenomic RNA known as the nucleocapsid (NC), which constitutes the biologically active template for both viral transcription and replication (Bloyet, 2021). The P protein functions as an indispensable cofactor for the viral polymerase L and acts as a molecular chaperone for NP during RNA synthesis (Zhou et al., 2025). In its free form, newly synthesized NP is prone to nonspecific binding to cellular RNA; however, P binds transiently to NP, maintaining it in an RNA-free, monomeric state that is competent for specific encapsidation of nascent viral RNA during replication (Bloyet, 2021). During transcription, the P protein tethers the L polymerase to the NP-encapsidated genomic template, facilitating processive RNA synthesis (Zhou et al., 2025). Moreover, P mediates the dynamic interaction between the polymerase complex and NC, enabling the polymerase to traverse the tightly packed NC structure without disassembling it (Blocquel et al, 2012; Whelan et al, 2004).

Building upon the essential roles of NP and P in genome encapsidation and polymerase activity, their physical interaction also results in the formation of viral inclusion bodies (IBs), prominent cytoplasmic structures observed during NDV infection (Wang et al, 2023). Historically, these IBs were first noted in histopathological examinations of infected tissues as eosinophilic aggregates and were long regarded as inert byproducts of viral protein overexpression or cellular stress responses (Li et al., 2023). However, recent advances in live-cell imaging, superresolution microscopy, and functional virology have fundamentally reshaped this view. These revealed that NDV-induced IBs are not merely pathological hallmarks but are also highly organized, functional compartments dedicated to viral genome replication and transcription (Dolnik et al, 2021). IBs serve as viral “replication factories” concentrating essential components, including the NP, P, L, and viral RNA, into discrete foci that optimize the efficiency and fidelity of the viral life cycle (Li et al., 2023). The IB formation begins early in infection, coinciding with the onset of viral gene expression (Wang et al, 2023). As newly synthesized NP and P accumulate in the cytoplasm, they coassemble into dynamic puncta that progressively mature into larger, microscopically visible IBs (Dolnik et al, 2021; Wang et al, 2023). Critically, the coexpression of NP and P alone without other viral proteins is sufficient to recapitulate IB-like structures in transfected cells, underscoring their role as the minimal molecular scaffold for IB formation (Nikolic et al, 2017). However, the specific residues that mediate the NP–P interaction are still unclear.

In this study, we demonstrated that both virulent and lentogenic strains of NDV induced IB formation in chicken-, mouse-, and human-derived cells. We further characterized the spatiotemporal dynamics and subcellular localization of IBs during infection. Moreover, we identified the NP–P binding sides and revealed that this interaction mediates IB-like structure formation.

## 2 Materials and methods

### 2.1 Ethics statement

All the animal experiments were approved by the Animal Care and Use Committee of the Northwest A&F University, China, and were conducted in accordance with guidelines established by the Chinese Committee for Animal Experiments (approval numbers: 2020ZX10010009–001 for chickens, 2020ZX06008011–003 for mice). Nine- to 11-day-old specific pathogen-free (SPF) embryonated chicken eggs were purchased from Jinan SAIS Poultry Co., Ltd. (China). The bleeding of the animals was performed under anesthesia via the use of ketamine and xylazine. All the mice were euthanized with carbon dioxide. All efforts were made to minimize suffering.

### 2.2 Viruses and cells

The velogenic strain F48E9 is used for standard vaccine challenge in China. The lentogenic strain LaSota is used globally for poultry vaccines. These viruses were propagated in 9- to 11-day-old SPF embryonated chicken eggs. All viruses were stored at -80°C. HEK293T cells (ATCC, CRL-11268), HeLa cells (ATCC, CCL-2), and DF-1 cells (ATCC, CRL-12203) were cultured in Dulbecco’s modified Eagle’s medium (DMEM) (Gibco, United States) supplemented with 10% fetal bovine serum (FBS) (Gibco, United States) and 1% Penicillin-Streptomycin (P/S) (Gibco, United States). Primary chicken embryo fibroblasts (CEFs) and mouse embryonic fibroblasts (MEFs) were prepared from 10-day-old SPF embryonated chicken embryos and embryonic day 13.5 (E13.5) mouse embryos as previously described (Batool et al, 2019; Wang et al, 2021).

### 2.3 Plasmid construction and transfection

The F48E9 NP, NP_1-375_, NP_1-366_, NP_1-357_, NP_1-320_, NP_33-489_, and NP (Q370A) were cloned and inserted into pcDNA3-HA. The F48E9 P, P ΔX domain, P ΔPMD domain, P ΔNP^0^-binding region, P ΔDisordered region, P ΔN terminal, and P (S370A) were cloned and inserted into pcDNA3-2×Flag. pCAGGS-myc-L was obtained from Shanhui Ren (Lanzhou Veterinary Research Institute). The primers used in this study are listed in **Table 1** and all the plasmids were confirmed by sequencing. The minigenome plasmid, containing an minigenome cassette (T7 promoter-Trailer-luciferase-Leader-HDV/Rbz-T7 terminator), and pCMV-NPL, expressing the LaSota NP, P and L proteins in tandem, and pCAGGS-T7 were constructed previously (Wang et al, 2019). The pCAGGS-T7, pCMV-NPL, and minigenome were cotransfected into DF-1 cells at a ratio of 2:1:1. Transfections were performed according to the protocol of the Turbofect transfection reagent protocol (ThermoFisher Scientific, United States).

**Table 1:** Primers of construction of.

Target	5’→3’ sequence^a,b^
NP	F: *GGATCC*ATGTCTTCCGTATTTGAT
	R: *GAATTC*TCAATACCCCCAGTCGGT
NP_1-375_	F: *GGATCC*ATGTCTTCCGTATTTGAT
	R: *GAATTC*ATTAATGCTACTTCCCT
NP_1-366_	F: *GGATCC*ATGTCTTCCGTATTTGAT
	R: *GAATTC*ATACTCTACTCCAAGT
NP_1-357_	F: *GGATCC*ATGTCTTCCGTATTTGAT
	R: *GAATTC*TGTGCTCATAAAGTCTCT
NP_1-320_	F: *GGATCC*ATGTCTTCCGTATTTGAT
	R: *GAATTC*CATTTGGTCACTGTCA
NP_33-489_	F: *GGATCC*TTGAAGTCCCAGTATT
	R: *GAATTC*TCAATACCCCCAGTCGGT
P ΔNP^0^-binding region	F: *GGATCC*CCATCGGCCAGCCAAGACACC
	R: *GAATTC*TCAGCCATTCAGCGCA
P ΔDisordered region	F: *GGATCC*AAAAAGGGCCCATGGTT
	R: *GAATTC*TCAGCCATTCAGCGCA
P ΔN terminal	F: *GGATCC*GCGATGATGTCTATGATGGA
	R: *GAATTC*TCAGCCATTCAGCGCA

^a^ F, forward primer; R, reverse primer.

^b^ Restriction enzyme sites are italic.

### 2.4 Viral titration

CEF, MEF, and HeLa cells were infected with F48E9 at multiplicities of infection (MOI) of 0.1 at 37°C for 1 h. Then, the cells were washed three times with sterile PBS and cultured in DMEM supplemented with 2% FBS and 1% P/S. The culture medium was harvested at different hpi. The CPE was visualized under an Olympus IX73 inverted research microscope (Japan). The viral titers were determined via a 50% tissue culture infective dose (TCID_50_/mL) assay and calculated according to the method established by Reed and Muench (Wang et al., 2017).

### 2.5 Immunofluorescence assay

CEFs, MEFs, HeLa and DF-1 cells were infected with F48E9 or LaSota strain at an MOI of 0.1 and 1 at 37°C for 1 h, respectively. Afterward, the cells were washed three times with sterile PBS and cultured in DMEM supplemented with 2% FBS. Virus-infected cells at 24 h post-infection (hpi) or transfected cells after 36 h were fixed with 4% paraformaldehyde in PBS for 15 min, washed three times with PBS, and permeabilized with 0.1% Triton X-100 for 10 min at room temperature. After blocking with PBS containing 1% bovine serum albumin (BSA) for 1 h, the cells were incubated with the primary antibodies anti-NP (LaSota) mouse pAb (1:200), anti-P (LaSota) guinea pig pAb (1:200) or anti-L (LaSota) guinea pig pAb (1:200) (Wang et al., 2019) in PBS at 4°C overnight. After being washed with PBS three times, the samples were incubated with the secondary antibodies goat anti-mouse IgG H&L (Alexa Fluor^®^ 488, preadsorbed, or Alexa Fluor^®^ 647, preadsorbed) (Abcam, United States) and goat anti-guinea pig IgG H&L (Alexa Fluor^®^ 488, preadsorbed, or Alexa Fluor^®^ 647, preadsorbed) (Abcam, United States) for 1 h at room temperature. The cell nuclei were stained with 4’,6’-diamidino-2-phenylindole (DAPI) (Beyotime Biotechnology, China). All the cells were observed and photographed under a confocal microscope (Olympus FV3000, Japan).

### 2.6 Coimmunoprecipitation

HEK293T cells in 60-mm-diameter dishes were cotransfected with two plasmids at a dose of 2 μg. The cells were lysed in 500 μl of ice-cold RIPA buffer supplemented with the protease inhibitor phenylmethylsulfonyl fluoride (PMSF; 1:1,000) (Solarbio, China) for 30 min at 36 h post transfection. After centrifugation at 13,000×g for 15 min at 4°C, 40 μl of the supernatant as the input sample was treated with 10 μl of 5×SDS loading buffer, and the remaining supernatants were incubated with DYKDDDK tag recombinant antibody (Proteintech, China) or HA tag pAb (Proteintech, China) at 4°C for 4 h. The immunoprecipitated complexes of every sample were captured by the addition of 50 μl of protein A/G-agarose (Abmart, China) to the slurry and gentle rotation overnight at 4°C. The agarose beads were further washed three times on ice, resuspended in 50 μl of 1×SDS loading buffer, and boiled for 10 min. After centrifugation, the supernatants were subjected to SDS–PAGE for western blot analysis.

### 2.7 Western blotting

The supernatants were subjected to 12.5% SDS–PAGE (Epizyme Biotech, China). The proteins were transferred to a polyvinylidene fluoride (PVDF) membrane (Immobilon^®^-P, United States). Immunoblotting was performed with the primary antibodies: DYKDDDDK tag mouse mAb (Proteintech, China), HA tag mouse mAb (Proteintech, China). The secondary antibody was horseradish peroxidase (HRP)-conjugated goat anti-mouse IgG (H+L) (Proteintech, China). Chemiluminescence (ECL) peroxidase substrate (Millipore, United States) was used for the detection of proteins via the Tanon 5200 Chemiluminescent Imaging System (Tanon, China).

### 2.8 Transmission electron microscopy

NDV-infected CEFs, MEFs, and HeLa cells at 24 hpi were fixed with 2.5% glutaraldehyde (Servicebio, China). TEM and ultrastructural analysis were performed by Wuhan Servicebio Technology Co., Ltd.

### 2.9 Measurement of IBs

The cross-sectional areas of IBs in virus-infected cells were measured by ImageJ. The size of IBs was represented by the area of IB within positive cells. Large, medium, and small IBs were quantified in randomly selected 200 IBs for each group (**Table 2**). The percentages of different-sized IBs were calculates in the total IB. Values were mean ± standard deviations (SD) from three experiments.

**Table 2:** Category of IB according to its size.

Category	Size (mean cross-sectional area)
Large	Greater than 8 μm^2^
Medium	Between 3 μm^2^ and 8 μm^2^
Small	Less than 3 μm^2^

### 2.10 Structural modeling and interface residue analysis

The nucleoprotein (NP, AVA10055.1, 489 aa) and phosphoprotein (P, AVA10056.1, 395 aa) sequences of NDV strain F48E9 were retrieved from the complete genome record MG456905.1 and verified against the independent records GQ370814.1/ACU81100.1 and AF327300.1/AAG60695.1. The full-length sequences were submitted to the HDOCK server (job 6a7b71f42a3f7) for global protein–protein docking without any predefined binding site, and the 20 returned models were postprocessed in Python with Biopython (Yan et al, 2020): a contact was defined as a residue pair whose closest heavy atoms on the two chains lie within 5.0 Å, duplicate residue pairs were collapsed, and contacts were filtered against the experimentally implicated regions NP 367–375 and P 226–294. Contacts within these regions were further classified into hydrogen-bond candidates, salt bridges and hydrophobic contacts on the basis of donor–acceptor chemistry, side-chain charge and side-chain carbon distances and were rendered with PyMOL. An independent cross-check was performed by docking the experimental NP structure 6JC3 chain A against the experimental P structure 7YOV chain E under the same contact criteria, with template-frame P numbering converted to full-length F48E9 numbering (offset-4, confirmed over 142 of the 158 modeled residues of chain E). For conservation analysis, all NDV NP records of 400–600 aa were downloaded from NCBI Protein (1614 entries); each record was anchored to F48E9 using the 12-residue sequence context flanking positions 367–375 (allowing up to two substitutions per flank) so that equivalent positions were compared even in the presence of indels elsewhere, and the 1610 successfully anchored sequences were used to compute per-position amino acid frequencies and to draw an information-content (bits) sequence logo with logomaker (Tareen and Kinney, 2020).

### 2.11 Statistical analysis

Two-tailed Student’s *t* tests were used to estimate the statistical significance between two columns in GraphPad Prism 5. Data from three independent experiments are presented as the means ± standard deviations (SDs) from triplicate samples (n≥3).

## 3 Results

### 3.1 NDV infection induces the formation of cytoplasmic IBs cross species

To examine the ultrastructural properties of the NDV IBs, the standard TEM of the infected cells was performed. CEFs, MEFs and HeLa cells were infected with F48E9 and LaSota at an MOI of 0.1 and 1, respectively. The cells were fixed for TEM analysis at 24 hpi. IBs were membraneless and located in the cytoplasm with high electron density in infected chicken and mammal cells (**Figure 1A**). And the virions were clearly observed in infected CEFs (**Figure 1A**). And the proliferation curves of F48E9 were measured (**Figure 1B**), showing that NDV could replicate in all of these cells, but the viral replication ability in MEF cells was significantly lower than that in other cells. To confirm that the IBs were induced by NDV infection, CEFs, MEFs and HeLa cells were infected with F48E9 (MOI = 0.1) and examined by IFA at 24 hpi. IBs were readily observed in avian and mammal cells, especially HeLa cells (**Figure 2A**). During F48E9 infection, the different sizes of IBs were essentially consistent between CEFs and HeLa cells, whereas F48E9 induced a greater number of small IBs in MEF cells compared to CEFs and HeLa cells (**Figure 2B**). These results indicated that NDV induced the formation of membraneless cytoplasmic IBs across species.

**Figure 1:**
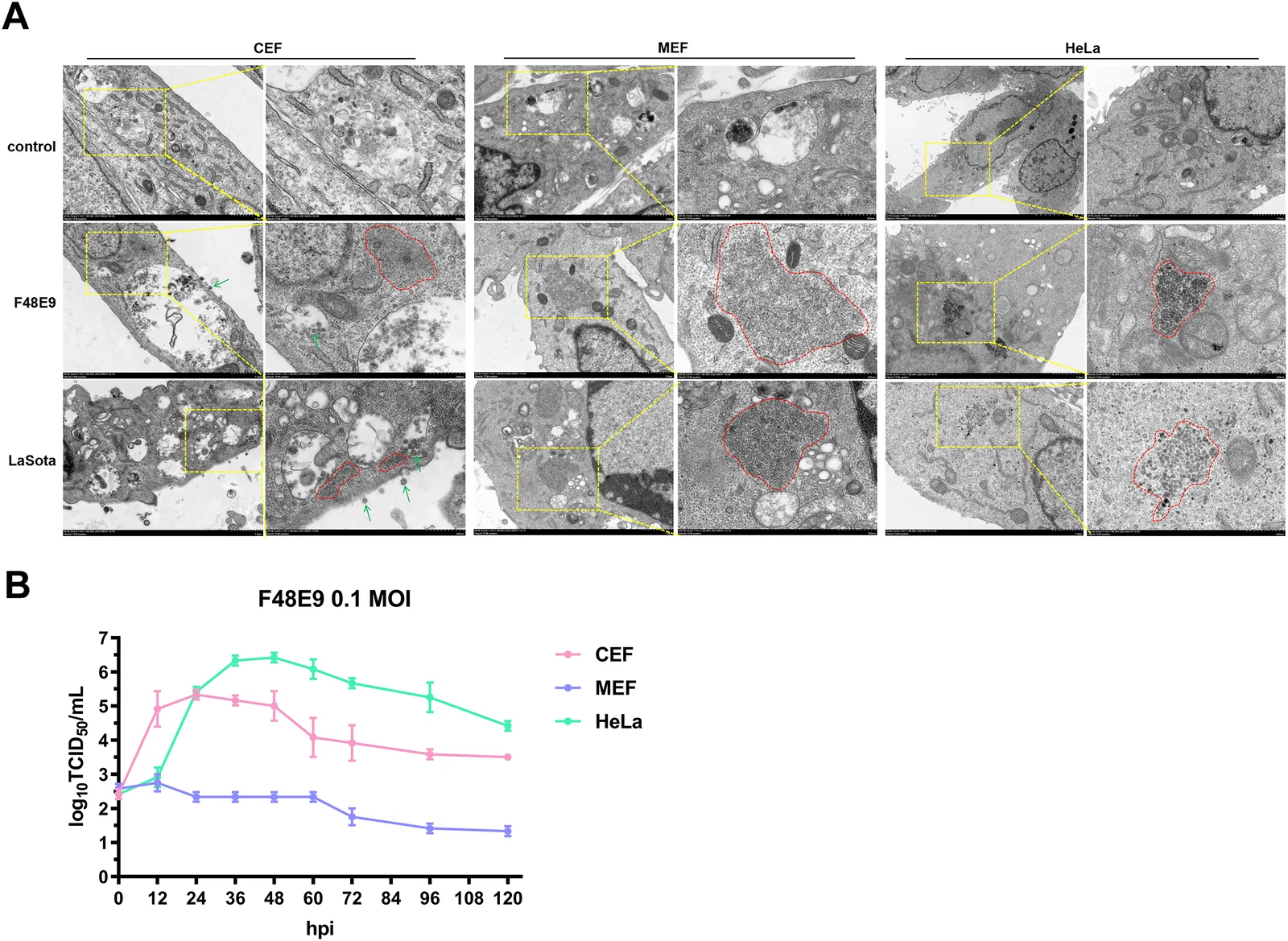
IBs in NDV-infected cells observed by TEM and viral replication in cells. **(A)** CEFs, MEFs and HeLa cells were infected with F48E9 and LaSota at an MOI of 0.1 and 1, respectively. At 24 hpi, the cells were fixed and processed for TEM. Low-magnification overview of a cell containing IBs (left). Scale bar=1.0 μm. Enlarged view of NDV IBs (yellow box on the right). NDV IBs are marked in red areas. Scale bar=500 nm. **(B)** CEFs, MEFs and HeLa cells were infected with F48E9 at an MOI of 0.1, respectively. The culture medium was harvested at different hpi. The viral titers were determined via TCID_50_/mL assay and calculated according to the method established by Reed and Muench. Representative data from three independent experiments, shown as the means ± SDs (n=3), were analyzed by two-tailed Student’s *t* tests.

**Figure 2:**
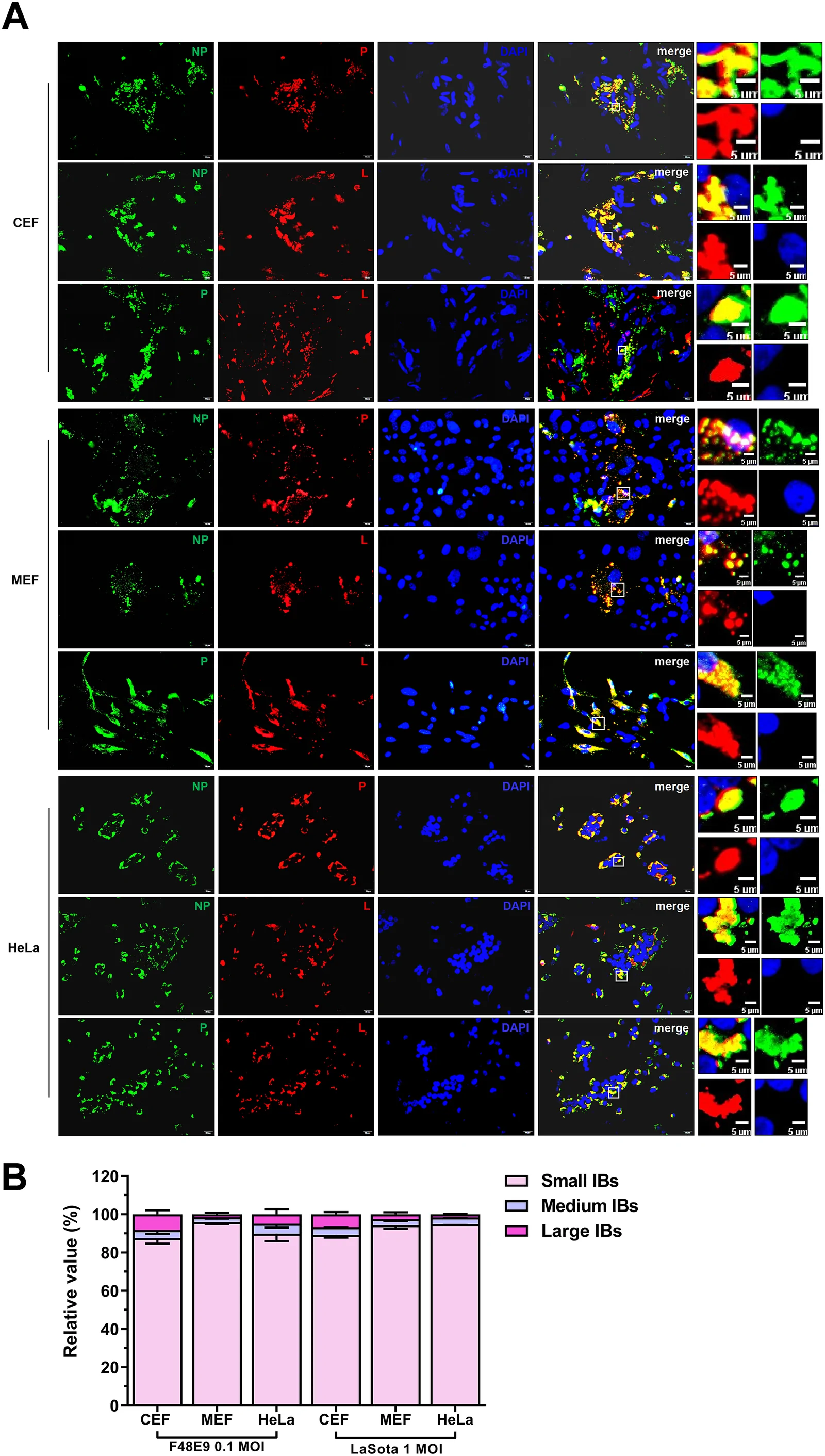
IBs in NDV-infected cells observed by IFA. **(A)** Localization of NDV NP, P, and L in F48E9-infected CEF, MEF and HeLa cells at 24 hpi by IFA with anti-NP (LaSota) mouse pAb, anti-P (LaSota) guinea pig pAb or anti-L (LaSota) guinea pig pAb. Scale bar=20 μm. The white box depicts the colocalization. Scale bar=5 μm. **(B)** Quantifications of large, medium and small IBs relative to IBs of all sizes in F48E9-infected cells at 24 hpi. The cross-sectional areas of the IBs in **(A)** were measured. Large, medium, and small IBs were quantified in randomly selected 200 IBs for each group. The graph shows the percentages of large, medium and small IBs to IBs of all sizes. Representative data from three independent experiments, shown as the means ± SDs (n=3).

### 3.2 Temporal and spatial distribution of IBs

As shown in **Figure 2**, NDV infection induced highly uniform and morphologically well-defined IBs in HeLa cells, making IBs easy to observe and measure size. Therefore, HeLa cells were used as a cellular model for spatiotemporal distribution analysis. NP and P were stained in F48E9-infected HeLa cells at 0, 3, 6, 9, 12, 24 hpi. IBs were first appeared around the nucleus at 6 hpi with small dispersed spherical puncta in most of the infected cells (**Figure 3**). As the time of infection increased, the size of puncta increased and their shapes became irregular (**Figure 3A**). At 12 hpi, the IBs were primarily of medium size (**Figure 3B**). At 24 hpi, IBs became fused and enlarged (**Figure 3**). The IBs were always located around the nucleus (**Figure 3A**). The size of IBs gradually increases and accumulates around the cell nucleus over time.

**Figure 3:**
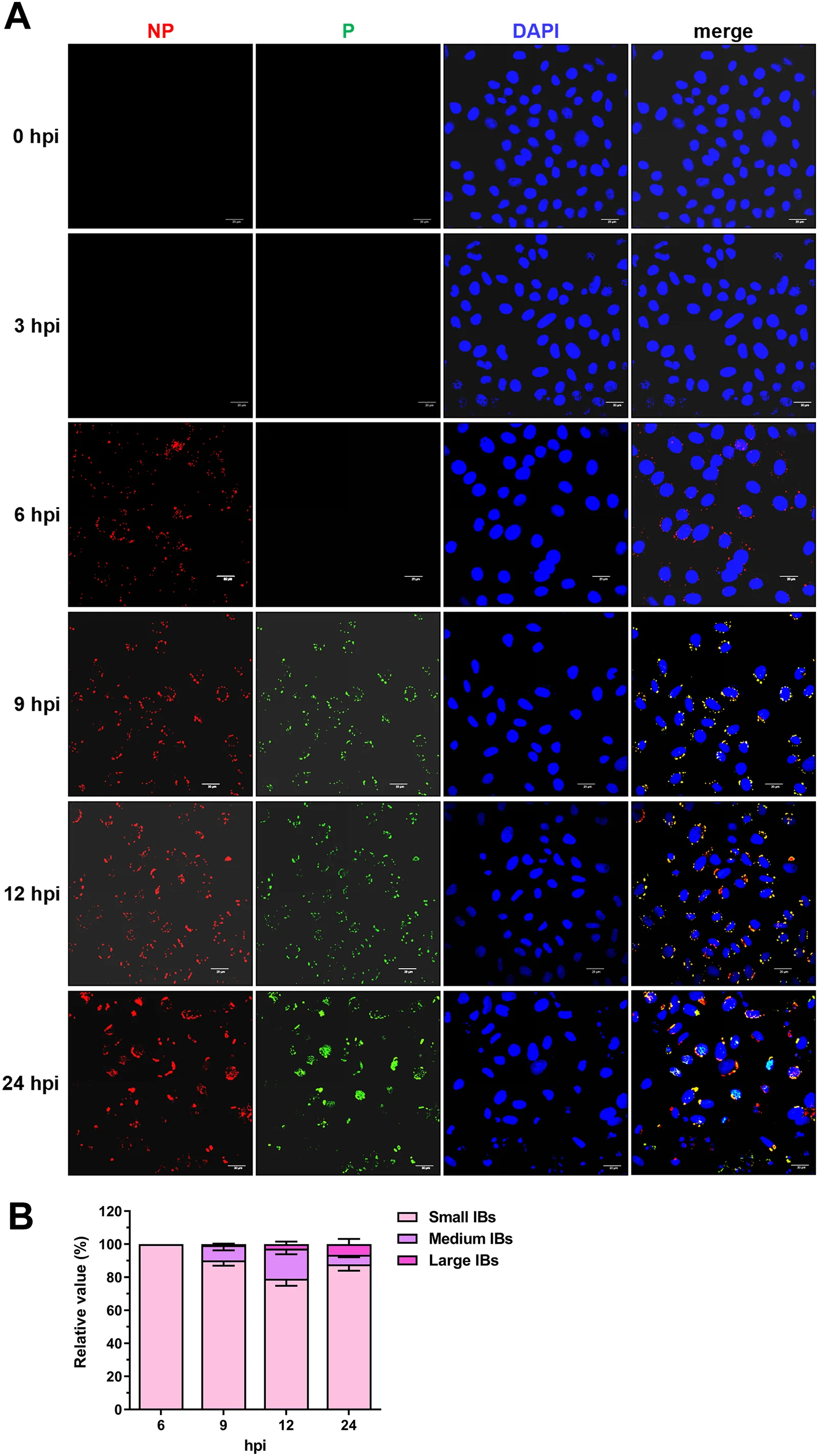
IB formation in NDV-infected HeLa cells observed by IFA. **(A)** HeLa cells were infected with F48E9 at an MOI of 0.1, and observed at different hpi by IFA with anti-NP (LaSota) mouse pAb and anti-P (LaSota) guinea pig pAb. Scale bar=20 μm. **(B)** The percentages of large, medium and small IBs to IBs of all sizes at 6, 9, 12, and 24 hpi. Representative data from three independent experiments, shown as the means ± SDs (n=3).

### 3.3 NP could induce IB-like structures

IBs serve as viral “replication factories” concentrating essential components, including NP, P, L, and nascent viral RNA, into discrete foci that optimize the efficiency and fidelity of the viral life cycle (Li et al., 2023). To recapitulate NDV pathogenicity *in vitro*, DF-1 cells, an avian-derived, highly permissive target cells, were employed to determine the minimal viral components involved in NDV IB formation. The viral proteins or DNA were transfected separately or in combination into DF-1 cells. When proteins were expressed alone, NP formed aggregates, particularly around the nucleus, and the P and L proteins were evenly distributed in the cytoplasm and did not form aggregates (**Figure 4A**). When NP and P, NP and L, P and L, or NPL were coexpressed, only the presence of NP induced IB-like structures (**Figures 4B, C**). To verify the function of viral RNA in IB formation, a minigenome plasmid was transfected into DF-1 cells with pCAGGS-NPL and pCAGGS-T7. IB-like puncta could form regardless of the presence of RNA (**Figure 4D**). These results suggested that the NP could induce IB-like structures.

**Figure 4:**
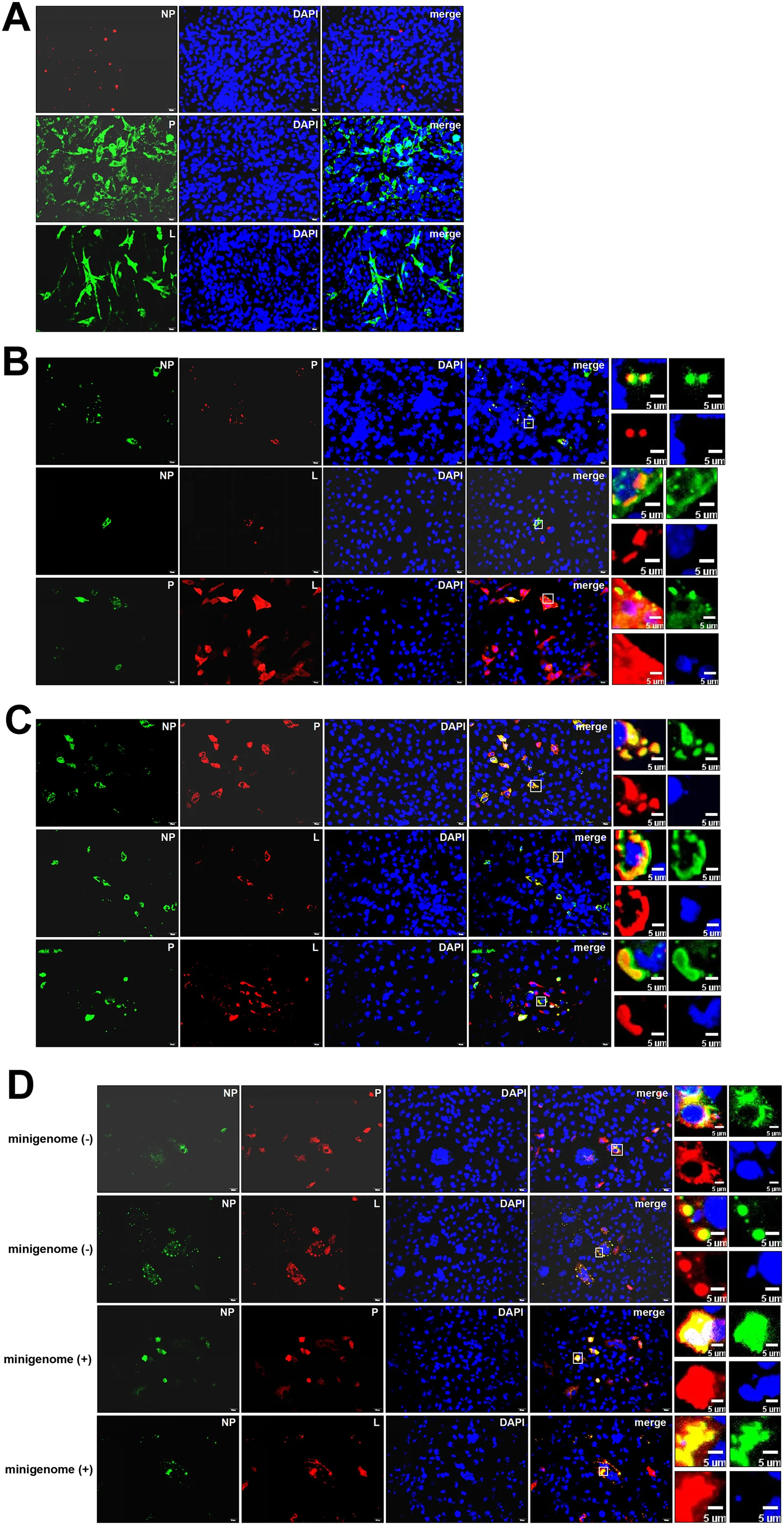
Viral NP protein is essential for the formation of IBs. DF-1 cells were transfected with pcDNA3-HA-NP, pcDNA3-2×Flag-P, and pCAGGS-myc-L individually **(A)**, with pairs **(B)**, or with three plasmids **(C)** or transfected with the minigenome plasmid and pCMV-NPL **(D)**. The cells were observed at 36 h post transfection by IFA with anti-NP (LaSota) mouse pAb, anti-P (LaSota) guinea pig pAb, and anti-L (LaSota) guinea pig pAb. Scale bar=20 μm. Enlarged view of NDV IBs (white box on the right). Scale bar=5 μm.

### 3.4 Residues 367–375 of NP interacting with the PMD of P is critical for the formation of IB-like structures

To investigate the region of NP required for IB-like structure assembly, we generated a series of N- and C-terminal truncations of NP and tested their ability to form IBs when they were coexpressed with P (**Figure 5A**). NP can be divided into several subdomains: the N-arm domain (NAD), the N-core region (NCR) composed of the N-terminal domain (NTD) and the C-terminal domain (CTD), and an intrinsically disordered C-terminal region (CTR) (Nath et al, 2020). The interaction was identified by co-IP. A C-terminal truncation assay revealed that deletion up to residue 375 (NP_1-375_) supported interaction with P and efficient IB-like structure formation, whereas further truncation to residue 366 (NP_1-366_) completely abrogated IB-like structure formation, resulting in a diffuse staining (**Figures 5B, C**). Deletion of the N-terminal 32 amino acids did not abolish IB-like structure assembly, and the NP–P interaction (**Figures 5B, C**). Correspondingly, P was divided into four regions (NP^0^-binding region, disordered region, PMD, and X domain) according to a previous report (Qiu et al, 2016). To identify which domain of P interacts with NP to induce IB-like structure formation, we constructed P truncations (**Figure 6A**). Co-IP and IFA assays revealed that the PMD of P interacting with NP was essential for the formation of IB-like puncta (**Figures 6B, C**). These results demonstrated that residues 367–375 of NP were critical binding residues with PMD of P which is vital for IB-like structure formation.

**Figure 5:**
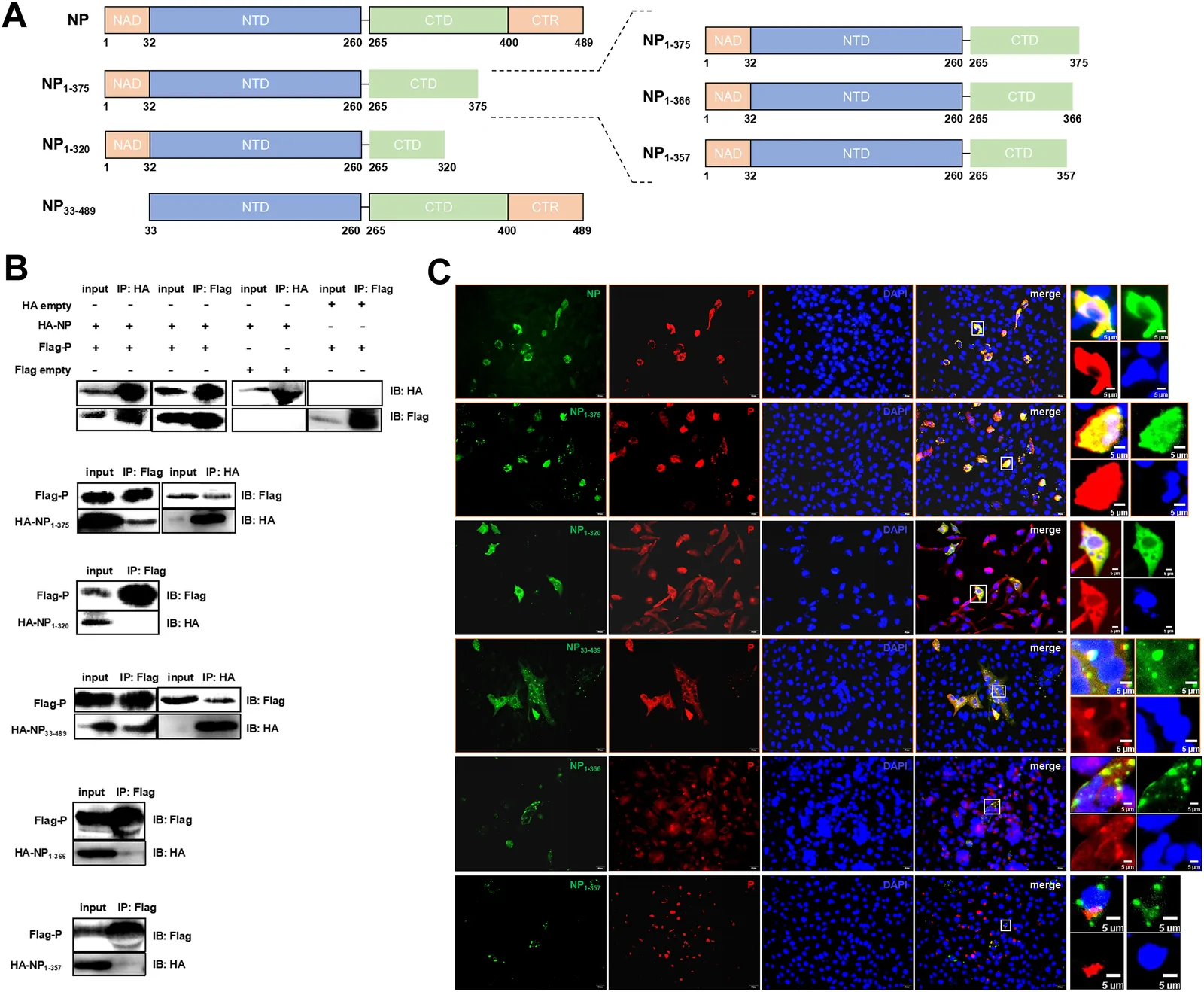
Interaction between NP truncations and (P)**(A)** Schematic representation of NP truncations. The domains are shown as different colored boxes. The amino acid size is depicted below the boxes. The abbreviation of each truncation is shown to the left of each schematic representation. **(B)** The interactions between the truncations of NP and P of the F48E9 strain were validated via co-IP assays. HEK293T cells were transfected with pcDNA3-HA-NP truncation plasmids and pcDNA3-2×Flag-P. Empty plasmids were used as negative controls. All the cell lysates were prepared at 36 h post transfection, and the proteins were immunoprecipitated via an anti-Flag rabbit recombinant antibody or an anti-HA rabbit pAb. The immunoprecipitated proteins were analyzed by western blotting. **(C)** Localization of NP truncation and P in the transfected DF-1 cells at 36 h post transfection by IFA with anti-NP (LaSota) mouse pAb, and anti-P (LaSota) guinea pig pAb. Scale bar=20 μm. The white box depicts the colocalization. Scale bar=5 μm.

**Figure 6:**
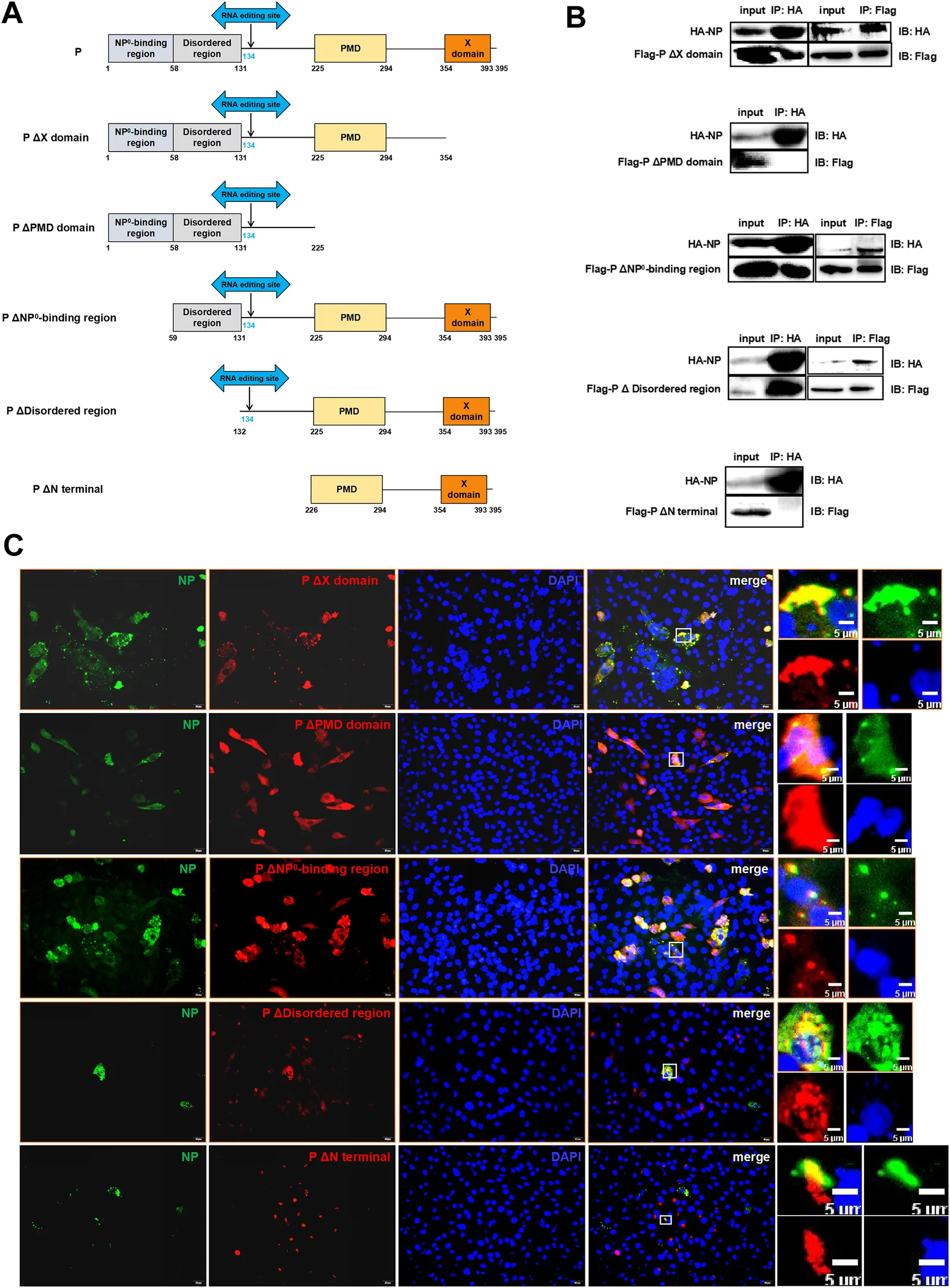
Interaction between P truncations and NP. Schematic representation of P truncations. The domains are shown as different colored boxes. The amino acid size is depicted below the boxes. The abbreviation of each truncation is shown to the left of each schematic representation. **(B)** The interactions between the P protein truncations and NP were validated via co-IP assays. HEK293T cells were transfected with pcDNA3-2×Flag-P truncation plasmids and pcDNA3-HA-NP. All the cell lysates were prepared at 36 h post transfection, and the proteins were immunoprecipitated via an anti-Flag rabbit recombinant antibody or an anti-HA rabbit pAb. The immunoprecipitated proteins were analyzed by western blotting. **(C)** Localization of P truncation and NP in the transfected DF-1 cells at 36 h post transfection by IFA with anti-NP (LaSota) mouse pAb, and anti-P (LaSota) guinea pig pAb. Scale bar=20 μm. The white box depicts the colocalization. Scale bar=5 μm.

### 3.5 Gln370 of NP and Ser254 of P are the interaction sites between NP and P for IB-like structure assembly

NP was identical to its independent record across all 489 aligned positions and P was 99.49% identical to its record, with both differences lying outside P 226–294, confirming that the experimentally implicated segments ^367^AQAQGSSIN^375^ of NP and residues 226–294 of P were stable between records. Global docking returned 20 models whose overall scores and regional coverage were discordant: Model 1 achieved the most favorable HDOCK score (–274.14) but formed only one contact inside the predefined regions, whereas Model 6 (–247.20) formed 7 clustered contacts there and was taken as the region-consistent representative conformation (**Figure 7A**). Its contacts involved only NP 369–371 and P 254–257 (**Supplementary Table 1**) and atom-level classification identified a single hydrogen-bond candidate, between NP Gln370 OE1 and P Ser254 OG at 2.66 Å (**Supplementary Table 2**), meeting the geometric criteria for hydrogen bonding candidates (**Figures 7B, C**). The hydrophobic P-side residues Ile255, Pro256 and Met257 faced the polar Gln370 and the side-chain-free Gly371, and thus contributed to van der Waals packing and main-chain shape complementarity rather than paired hydrophobic interactions (**Supplementary Table 2**). Finally, alignment of the 1610 NDV NP sequences revealed that NP 367–375 was nearly invariant in nature, with every position retaining its consensus residue in at least 97.64% of the strains and the consensus matching the F48E9 sequence at all nine positions; notably, the three interface residues were among the most conserved in the window, with Gln370 conserved in 99.81% of the strains, Ala369 in 99.94%, and Gly371 completely invariant across all 1610 sequences (**Supplementary Table 3**; **Figure 7D**). Subsequent co-IP and IFA assays clearly revealed that the mutation of NP Gln370 or P Ser254 significantly impaired the NP–P interaction and disrupted the formation of IB-like structures (**Figure 7E, F**). Together, these findings collectively validated that NP Gln370 and P Ser254 are critical residues essential for IB-like structure assembly.

**Figure 7:**
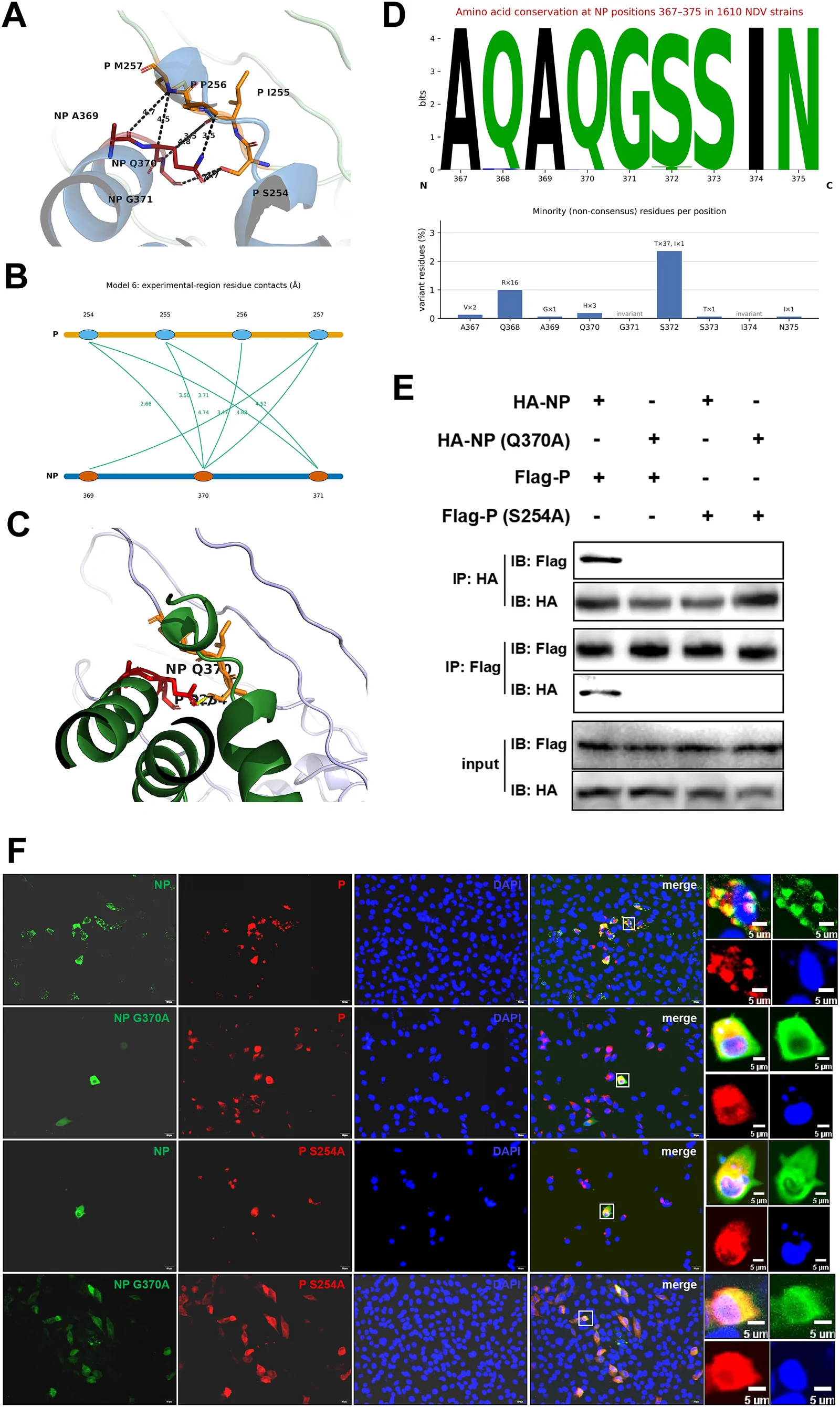
Structural modeling and interface residue analysis of the NP–P interaction in NDV strain F48E9. **(A)** Three-dimensional contact view of model 6. Interface residues are drawn as sticks and yellow dashed lines indicate the contact geometry. **(B)** Two-dimensional residue contact diagram of model 6. P 254–257 is shown above and NP 369–371 below, with the shortest atomic distance annotated beside each connecting line. **(C)** Labelled key-residue figure of model 6. NP Q370 and P S254 are labelled, and the yellow dashed line marks the Gln370 OE1–Ser254 OG hydrogen-bond candidate. **(D)** Amino acid conservation at NP 367–375 across 1610 NDV strains. The upper panel is a sequence logo on an information-content (bits) scale, in which the letter height reflects the degree of conservation at each position; the lower panel magnifies the proportion of nonconsensus residues per position and annotates the specific substitutions and their strain counts. **(E)** The interactions between NP Q370 mutation and P or P S254 mutataion and NP were validated via co-IP assays. The immunoprecipitated proteins were analyzed by western blotting. **(F)** Localization of NP Q370 mutation and P or P S254 mutataion and NP in the transfected DF-1 cells at 36 h post transfection by IFA with anti-NP (LaSota) mouse pAb, and anti-P (LaSota) guinea pig pAb. Scale bar=20 mm. The white box depicts the colocalization. Scale bar=5 mm.

## 4 Discussion

In this study, we investigated the formation, spatiotemporal dynamics, and molecular determinants of IBs induced by NDV. We demonstrated that both virulent and attenuated NDV strains triggered IB formation in chicken, mouse, and human cells. IBs grew from small puncta into larger structures that ultimately accumulated in the perinuclear region. We determined that residues 367–375 of NP bound to the PMD of P, with residue Gln370 of NP and residue Ser254 of P forming the interaction interface, which was necessary for IB-like structure formation (**Figure 8**). Together, these findings establish a conserved mechanism of IB formation and map a discrete NP–P binding motif in both virulent and attenuated strains.

**Figure 8:**
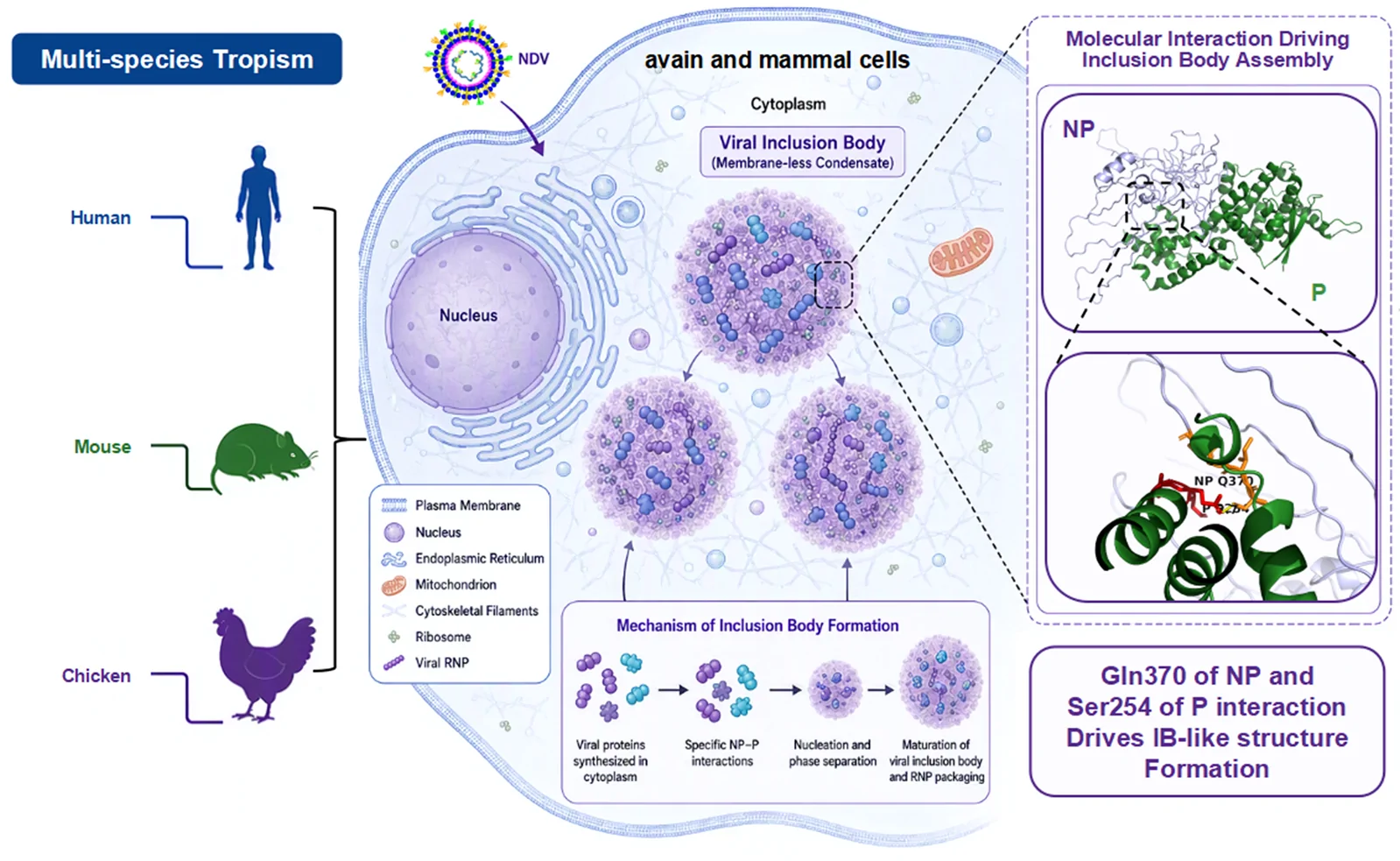
Schematic diagram of NDV IB formation across multiple host species.

The induction of IBs by both virulent and attenuated NDV strains, together with their formation across avian and mammalian cells, indicates that IB biogenesis is a conserved feature of NDV infection rather than a correlate of virulence or host adaptation. This is consistent with observations in PIV5 (Wignall-Fleming et al, 2024), and MeV (Zhou et al, 2019). IBs are now widely regarded as functional viral factories that concentrate the replication machinery: Negri bodies in rabies virus infection recruit all the components required for transcription and replication (Nikolic et al, 2017), and NDV IBs were previously shown to contain newly synthesized viral RNA and to exhibit liquid–liquid phase separation (LLPS) features (Wang et al, 2023). Notably, NDV replicated well in both avian and cancer cells. Its robust replication in chicken cells reflects the natural susceptibility of avian species, particularly chickens, as primary hosts for NDV. Importantly, NDV also exhibits oncolytic property, selectively infecting and replicating, and inducing lytic death of cancer cells while sparing normal mammal cells (Qu et al, 2026).

A central contribution of this study is the identification of a discrete NP–P binding interface that mediates IB formation. We previously demonstrated that NP and P are the minimal viral components required to generate IB-like puncta, and that the N arm and N core regions of NP, together with the C-terminus of P, contribute to this process (Wang et al, 2023). The present data refine this map by localizing a binding motif to residues 367–375 within the NP core and by assigning a previously unrecognized role to the PMD of P. Furthermore, Gln370 of NP and residue Ser254 of P were the interaction interface and necessary for IB-like structure formation. This contact is functionally distinct from the two canonical NP–P interactions described in paramyxoviruses: the N-terminal region of P, which chaperones unassembled NP (N^0^) to prevent premature oligomerization, and the C-terminal X domain of P, which binds the NP tail of the assembled N–RNA template to position the polymerase (Bloyet, 2021). Because the PMD mediates P oligomerization into tetramers, its engagement by NP provides multivalency; each P oligomer presents multiple binding surfaces for NP, and polymeric NP arrays present repeated 367–375 motifs. Such multivalent, weak-affinity interactions are precisely the biophysical drivers of LLPS (Banani et al, 2017), offering a mechanistic explanation for how a short linear motif within the NP core can nucleate phase separation in concert with the oligomeric P scaffold. In this regard, our findings complement rather than contradict the previously reported contribution of the P C-terminus, suggesting that multiple NP–P contact surfaces act cooperatively to stabilize the condensed phase. Although the interaction between Gln370 of NP and Ser254 of P was identified in this study, the structures of NP–P, or even the interaction structure of the NP–P–L complex need to be resolved via cryo-EM or X-ray crystallography, which will advance our understanding of the ribonucleoprotein (RNP) of paramyxovirus.

Our findings identified a newly NP-P binding interface, Gln370 of NP and Ser254 of P, that associated with IB-like structure formation, providing a structural basis for understanding condensate biogenesis in paramyxoviruses and a potential target for antiviral intervention.

## Data Availability

The original contributions presented in the study are included in the article/**Supplementary Material**. Further inquiries can be directed to the corresponding author.
