# Supplementary material for: Residue Gln370 of Newcastle disease virus nucleoprotein mediate viral inclusion body-like structure formation via interaction with residue Ser254 of viral phosphoprotein
Source: Front Cell Infect Microbiol. 2026 Oct 23;16:1978310. doi: 10.3389/fcimb.2026.1978310 (PMC13739580; doi:10.3389/fcimb.2026.1978310)
Supplement: Supplementary file 1 [file Table1.docx]

**Table 1. The 7 deduplicated residue contacts within NP 367–375/P 226–294 in HDOCK model 6.**

| **NP residue** | **P residue** | **Shortest atomic distance (Å)** | **Interface position** |
| --- | --- | --- | --- |
| Ala369 | Met257 | 4.74 | Peripheral contact |
| Gln370 | Ser254 | 2.66 | Core contact |
| Gln370 | Ile255 | 3.50 | Core contact |
| Gln370 | Pro256 | 3.47 | Core contact |
| Gln370 | Met257 | 4.52 | Peripheral contact |
| Gly371 | Ser254 | 3.71 | Core contact |
| Gly371 | Ile255 | 4.82 | Peripheral contact |

Core contacts are defined as a shortest heavy-atom distance ≤ 4.0 Å; the remainder are recorded as peripheral contacts.

**Table 2. Atom-level interaction classification of the experimental-region interface in model 6.**

| **Interaction type** | **Result** | **Representative residues / atoms** | **Distance** | **Structural implication** |
| --- | --- | --- | --- | --- |
| Hydrogen-bond candidate | 1 pair(s) | NP Gln370 OE1 – P Ser254 OG | 2.66 Å | Directional polar interaction at the interface core |
| Salt bridge | 0 pair(s) | No qualifying acid–base side-chain pair detected | — | No defined charge pairing |
| Hydrophobic contact | 0 pair(s) | No qualifying cross-chain hydrophobic side-chain pair detected | — | No paired hydrophobic interaction |
| General close contact | 7 residue pairs | NP 369–371 / P 254–257 | 2.66–4.82 Å | Forms a clustered spatial interface |

**Table 3. Per-position conservation of NP 367–375 across 1610 NDV strains.**

| **Position** | **F48E9 residue** | **Consensus** | **Consensus fraction** | **Variant residues (strains)** | **Structural role** |
| --- | --- | --- | --- | --- | --- |
| 367 | A | A | 99.88% | V (2) | — |
| 368 | Q | Q | 99.01% | R (16) | — |
| 369 | A | A | 99.94% | G (1) | Peripheral interface contact |
| 370 | Q | Q | 99.81% | H (3) | Hydrogen-bond donor/acceptor |
| 371 | G | G | 100.00% | None | Close main-chain packing |
| 372 | S | S | 97.64% | T (37); I (1) | — |
| 373 | S | S | 99.94% | T (1) | — |
| 374 | I | I | 100.00% | None | — |
| 375 | N | N | 99.94% | I (1) | — |

Of the 1614 NP records retrieved, 2 could not be anchored unambiguously and 2 contained non-standard characters; these were excluded.
